# A New Bifidobacteria Expression SysTem (BEST) to Produce and Deliver Interleukin-10 in *Bifidobacterium bifidum*

**DOI:** 10.3389/fmicb.2018.03075

**Published:** 2018-12-21

**Authors:** Aurélie Mauras, Florian Chain, Aurélie Faucheux, Pauline Ruffié, Sophie Gontier, Bernhard Ryffel, Marie-José Butel, Philippe Langella, Luis G. Bermúdez-Humarán, Anne-Judith Waligora-Dupriet

**Affiliations:** ^1^EA 4065, Ecosystème Intestinal, Probiotiques, Antibiotiques, Faculté de Pharmacie de Paris, DHU Risques et Grossesse, Université Paris Descartes, Paris, France; ^2^INRA, Commensals and Probiotics-Host Interactions Laboratory, Micalis Institute, AgroParisTech, Université Paris-Saclay, Jouy-en-Josas, France; ^3^UMR 7355 CNRS, Laboratory of Experimental and Molecular Immunology and Neurogenetics, University of Orleans, Orleans, France

**Keywords:** *Bifidobacterium bifidum*, microbiota, recombinant bacteria, heterologous expression system, IL-10, low-grade intestinal inflammation

## Abstract

In the last years there has been a growing interest in the use of genetically modified bacteria to deliver molecules of therapeutic interest at mucosal surfaces. Due to the well-recognized probiotic properties of some strains, bifidobacteria represent excellent candidates for the development of live vehicles to produce and deliver heterologous proteins at mucosal surfaces. However, very few studies have considered this genus because of its complexity to be genetically manipulated. In this work, we report the development of a new Bifidobacteria Expression SysTem (BEST) allowing the production of heterologous proteins in *Bifidobacterium bifidum*. This system is based on: i) the broad host range plasmid pWV01, ii) a stress-inducible promoter, and iii) two different signal peptides (SPs) one issued from *Lactococcus lactis* (SP_Exp4_) and issued from *Bifidobacterium longum* (SP_BL1181_). The functionality of BEST system was validated by cloning murine interleukin-10 (IL-10) and establishing the resulting plasmids (i.e., pBEST_Exp4_:IL-10 and pBEST_BL1181_:IL-10) in the strain of *B. bifidum* BS42. We then demonstrated *in vitro* that recombinant *B. bifidum* BS42 harboring pBEST_BL1181_:IL-10 plasmid efficiently secreted IL-10 and that this secretion was significantly higher (sevenfold) than its counterpart *B. bifidum* BS42 harboring pBEST_Exp4_:IL-10 plasmid. Finally, we validated *in vivo* that recombinant *B. bifidum* strains producing IL-10 using BEST system efficiently delivered this cytokine at mucosal surfaces and exhibit beneficial effects in a murine model of low-grade intestinal inflammation.

## Introduction

Bifidobacteria are strict anaerobic Gram-positive bacteria with irregular forms, belonging to the dominant microbiota in the human gut (especially in young children). Some species of bifidobacteria display beneficial properties for human health and are recognized as GRAS (Generally Regarded As Safe) microorganisms. In addition, some strains are already marketed as food supplements (with probiotic claims) such as *Bifidobacterium breve* M16-V, *Bifidobacterium longum* BB536, and *Bifidobacterium bifidum* BGN4. Probiotics are live microorganisms that, when administered in adequate amounts, confer a health benefit on the host, as defined by the World Health Organization (WHO) and the Food and Agriculture Organization of the United Nations (FAO) (Hill et al., 2014).

While recombinant bacteria are used in industry for the production of insulin or vaccines, researchers are increasingly interested by the direct administration of these recombinant bacteria to humans for either the development of mucosal vaccines or *in situ* delivery of proteins of therapeutic interest such as antioxidants, cytokines and protease inhibitors (Bermúdez-Humarán et al., 2013; Kumar et al., 2016). One of the main benefits in the use of probiotics as live vectors to deliver biologically active cytokines is to combine: (i) the intrinsic beneficial properties of some strains and (ii) the potential effect of the delivered protein. Moreover, several probiotics strains belong to some bacteria already present in our organism (i.e., intestinal microbiota) such as certain lactobacilli and bifidobacteria strains. This feature allows these strains to withstand gut environment wherein they can release biologically active molecules. Most bacteria used to deliver heterologous protein so far are lactobacilli and lactococci. Indeed, several trials have been carried out in animal models using either recombinant lactococci or lactobacilli to treat different human pathologies such as irritable bowel syndrome (IBS) and inflammatory bowel disease (IBD) (Motta et al., 2012; Martín et al., 2014; Allain et al., 2016). Strikingly, some human clinical trials (phase I and II) have been conducted with *L. lactis* producing either IL-10 or trefoil factor (TTF) to treat, respectively IBD (Braat et al., 2006) and oral mucositis (Limaye et al., 2013), confirming both the feasibility and the potential in the use of recombinant probiotics for human health.

In contrast to lactobacilli and lactococci, *Bifidobacterium* sp. could be a more attractive live vector to deliver proteins. Indeed, *B. breve* has been shown to persist for up to 32 days into mouse digestive tract after 3 days of gavage (Sheehan et al., 2007), in contrast to *Lactobacillus plantarum* which persists only 10 days (Pavan et al., 2003) and *Lactococcus lactis* that did not colonize with a transit time of 24–48 h (Kimoto et al., 2003). Finally, bifidobacteria have a low intrinsic and acquired resistance to antibiotics, showing therefore a safe profile for use in humans (Moubareck et al., 2005). Today, only few strains of bifidobacteria have been proposed as live delivery vehicles. For instance, some strains of *B. longum* have been used as a delivery vector for the development of: (i) oral vaccine against Hepatitis C virus (HVC) and Enterovirus 71 (Yu et al., 2013; Takei et al., 2014), (ii) anti-tumoral treatment (Shirakawa and Kitagawa, 2017), and (iii) to treat metabolic disorders in mice (Zhang et al., 2018). Other reports have described the use of genetically modified bifidobacteria to express diverse heterologous proteins (Khokhlova et al., 2010; Losurdo et al., 2013).

Currently, several expression systems are available for both lactobacilli and lactococci, including constitutive and inducible promoters and the possibility to express the desired protein into different cellular locations (i.e., intracellular, extracellular, or cell wall-attached) (Bermúdez-Humarán et al., 2013). However, as some proteins (such as cytokines) can accumulate in the cytoplasm and consequently be deleterious to bacteria growth, there is a need to develop new controlled-expression systems. These systems could avoid the negative outcomes of the over-production of heterologous proteins by controlling expression through an inductor such as changes in either the pH, temperature or even the addition of bile salts or antimicrobial peptides. One of the best-described expression systems in *L. lactis* is the NICE system (for NIsin Controlled Expression) (Kuipers et al., 1995), where gene expression is turned on by the addition of nisin to the culture medium. However, NICE system requires the presence of some regulatory genes (i.e., *nisRK*) that hampers its further applications in both fed-batch production and *in vivo* models. One alternative is the use of an expression system that does not require either the presence of regulatory genes or induction of bacterial cultures before use. Thus, we developed in our laboratory two *in vivo* stress-inducible expression systems for both lactococci [i.e., the SICE system (Benbouziane et al., 2013)] and lactobacilli [i.e., the LIVE system (Allain et al., 2016)], which allow the direct delivery of heterologous proteins *in situ* by recombinant bacteria. In the current work, we seek to develop a comparable system to deliver proteins of health interest by recombinant bifidobacteria. This system, named BEST for Bifidobacteria Expression SysTem, is based in the use of the broad-host range plasmid pWV01 (Morello et al., 2008), a *dnaK* promoter from *B. longum dnaK* operon, and two different signal peptides (SPs): one issued from the lactic acid bacterium (LAB) model *L. lactis* (SP_Exp4_) (Morello et al., 2008), and one from *B. longum*: SP_BL1181_. Furthermore, the functionality of BEST system was validated *in vivo*. For this, we cloned murine interleukin-10 (IL-10) an anti-inflammatory cytokine successfully expressed in different LAB (Braat et al., 2006), under BEST system and we determine its beneficial effects in a model of murine colitis (Martín et al., 2014). Our results show that genetically modified *B. bifidum* BS42 harboring IL-10 under BEST system successfully delivered the recombinant cytokine at mucosal surfaces and display beneficial properties in the murine model of intestinal low-grade inflammation.

## Materials and Methods

### Bacterial Strains, Plasmids, and DNA Manipulation

Strains and plasmids used in this work are listed in Table 1. *Escherichia coli* was grown in Luria-Bertani medium (Oxoid, Dardilly, France) at 37°C, under aerobic conditions with shaking. *L. lactis* was grown in M17 medium (Difco Laboratories, Detroit, MI, United States) supplemented with 1% glucose at 30°C without agitation. Bifidobacteria were cultured in de Man Rogosa Sharpe medium (MRS, Oxoid), supplemented prior to inoculation with cysteine (500 mg/L, Oxoid), at 37°C under anaerobic conditions (N2:CO2:H2; 80:10:10) in an anaerobic chamber (MACS, Chemunex AES Laboratoire, Bruz, France). Recombinant bacteria were selected by the addition of chloramphenicol (10 mg/L) except for *E. coli* harboring TOPO plasmid which was selected with kanamycin (50 mg/L).

**Table 1:** Bacterial strains and plasmids used in this study.

Strain or plasmid	Characteristic(s)^a^	Reference or source
**Strains**		
*E. coli* TOP10	Commercial competent cells	TOPOKit Invitrogen
*B. bifidum* BS42	Infant feces	Ménard et al., 2008
**Plasmids**		
pCR^®^2.1	Ap^R^K^R^ subcloning TOPO vector	TOPOKit Invitrogen
pCR-TOPO:*dnaK*	Ap^R^K^R^pCR-TOPO subcloning vector harboring a DNA fragment containing *dnaK* promoter	This study
pLB141	Cm^R^, pGK plasmid (a derivative from the broad host range plasmid pWV01) expressing a secreted form of the Staphylococcal nuclease (Nuc)(cassette SPexp4-NucB) under the control PnisA promoter	Morello et al., 2008
pLB270	Cm^R^, pGK plasmid expressing a secreted form of murine IL-10 (cassette SPLcob-IL-10) under the control PnisA promoter	Motta et al., 2012
pBEST_Exp4_:NUC	Cm^R^, pGK plasmid expressing a secreted form of the Staphylococcal nuclease (Nuc)(cassette SPexp4-NucB) under the control PdnaK promoter	This study
pBEST_Exp4_:IL-10	Cm^R^, pGK plasmid expressing a secreted form of murine Il-10 (cassette SP_Exp4_-IL-10) under the control PdnaK promoter	This study
pCR-TOPO:SP_BL1181_	Ap^R^K^R^pCR-TOPO subcloning vector harboring a DNA fragment containing the signal peptide of *BL1181* hypothetical protein from *B. longum*	This study
pBEST_BL1181_:IL-10	Cm^R^, pGK plasmid expressing a secreted form of murine IL-10 (cassette SP_BL1181_-IL-10) under the control PdnaK promoter	This study

*^a^For strains origin are given; for plasmids and cloned cassette characteristic are given. Ap, ampicillin; K, kanamycin; Cm, chloramphenicol.*

General procedures for DNA manipulation were essentially performed as described previously (Sambrook et al., 1989). Plasmid DNA isolation was performed using the Qiagen Midi-prep kit (Qiagen, Courtaboeuf, France). DNA sequences were confirmed by sequencing (Genewiz, Paris, France). Plasmid constructions were first established in either *E. coli* or *L. lactis* by electrotransformation (Sambrook et al., 1989) and then transferred into bifidobacteria. Electrocompetent *B. bifidum* protocols were adapted from Alvarez (Álvarez-Martín et al., 2008). Briefly, bifidobacteria were grown up to the optical density 0.6 at 600 nm (OD_600nm_) in MRS supplemented with 0.5 M of sucrose. Cultures were then put on ice for 20 min. After centrifugation for 15 min at 4700 rpm, 4°C, pellets were washed three times with cold 0.5 M sucrose + 10% glycerol. This step allowed a 200-fold bifidobacteria concentration. Aliquots were incubated 1 h on ice, and then frozen at -80°C. Cells (5 × 10^10^ colony-forming units, CFU/mL) were electroporated at 200 Ω, 2.5 kV/cm, 25 μF (Bio-Rad, Marnes-la-Coquette, France) with 0.1–0.3 μg of DNA and resuspended in MRS for 3 h at 37°C in anaerobic condition before plating on MRS agar plus chloramphenicol (10 mg/L).

### Construction of the Bifidobacteria Expression SysTem (BEST)

**FIGURE 1:**
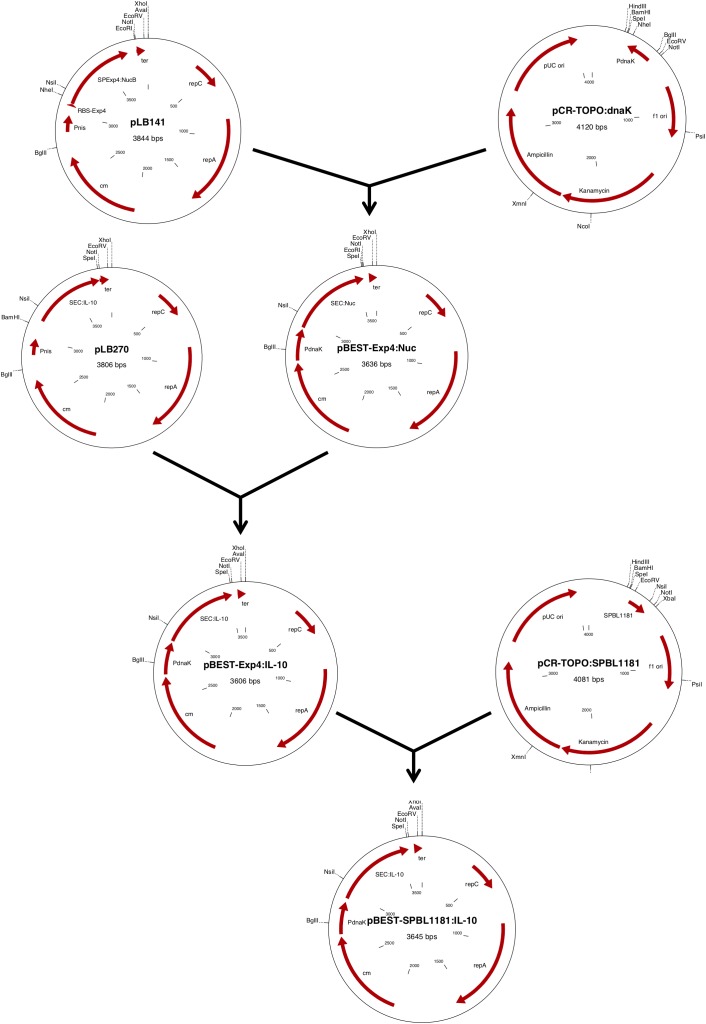
Schematic representation of plasmids construction.

For the construction of a vector allowing heterologous protein production in *Bifidobacterium* sp., we cloned *dnaK* promoter from a strain of *B. longum* into pLB141 vector (Table 1; Morello et al., 2008), a derivative of the broad-host range plasmid pWV01 (GenBank Accession Number: NC_002192). Briefly, a DNA fragment (241 bp) containing *dnaK* promoter was amplified by PCR from genomic DNA of *B. longum* (GenBank Accession Number: NC_004307.2) with primers: *Bgl*II-P*dnaK* (5′-CCA***AGATCT***CAAAAAACBTGAGCCWAMMWYRCTCAACTT-3′) for the coding strand and *Nhe*I-P*dnaK* (5′-***AGCT*** ***AGC***GTTAATAAAGCAAGGTTTATTTTTTTCATAACGTGTGCTCCTTAATTAYTCGTTTGTTCTTACGTTSTTYG-3′) for the complementary strand (with B = C or G or T; W = A or T; M = A or C; Y = C or T; R = A or G). The PCR product was then subcloned into a pCR-TOPO plasmid (Invitrogen, Carlsbad, CA, United States) resulting in pCR-TOPO:*dnaK* (Table 1). Then, the DNA fragment containing *dnaK* promoter was recovered from pCR-TOPO:*dnaK* plasmid with *Bgl*II/*Nhe*I enzymes (New England Biolabs, Evry, France) and cloned into purified backbone isolated from *Bgl*II/*Nhe*I-cut pLB141 vector, resulting in pLB141:*dnaK*:Nuc. Nucleotide sequence analysis of this construction revealed a deletion in the SP_Exp4_ (data not shown). To repair this, we performed a directed mutagenesis step by PCR. Briefly, we established pLB141*:dnaK*:Nuc vector in *E. coli* TG1 strain in order to purify large quantities of the plasmid, we then performed a first PCR reaction with forward primer: 5′-GTTATGAAAAAAATAAACCTTGCTTTATTAACGCTA-3′ using *Pfu* turbo DNA polymerase (Promega). Parameters of PCR were a first cycle at 95°C 1 min and 5 cycles at 95°C 30 s, 55°C 1 min, and 68°C 4 min. Afterward, we performed a second PCR reaction on the first PCR product with forward and reverse primers: 5′-TAGCGTTAATAAAGCAAGGTTTATTTTTTTCATAAC-3′). PCR parameters were as follow: first cycle 95°C, 1 min; 20 cycles at 95°C 30 s/55°C 1 min/68°C 4 min; 4°C. Then we performed one digestion with *Dpn*I enzyme (New England Biolabs, Evry, France) before establishment of the plasmid into *L. lactis*. The final construction pBEST_Exp4_:Nuc (Figure 1 and Table 1) was confirmed by sequencing.

### Expression of IL-10 Cytokine Using BEST System

For the expression of IL-10 using BEST system, a DNA fragment encoding for IL-10 mature sequence was obtained from a plasmid harboring murine *il-10* gene (cassette SP_Usp45_:IL-10) under the control of P*_nisA_* promoter, also known as pLB270 plasmid (Motta et al., 2012; Benbouziane et al., 2013), with *Nsi*I/*Spe*I enzymes (Thermo Scientific, Courtaboeuf, France) and cloned into pBEST_Exp4_:Nuc vector digested with the same enzymes and replacing the corresponding *nuc* DNA fragment. The resulting vector pBEST_Exp4_:IL-10 was established into *B. bifidum*.

In parallel and in order to determine the impact of SP_Exp4_ (issued from *L. lactis*) for heterologous protein secretion in bifidobacteria, we replaced this SP with a new one issued from *B. longum*: SP of the hypothetical protein BL1181 from *B. longum* NCC2705 (Schell et al., 2002). Indeed, after *in silico* analysis (using SignalP software^1^) of homologous proteins efficiently secreted by bifidobacteria we put a particular interest in this SP since it was one with the highest score of “cleavage site.” For the cloning: a DNA fragment containing the SP of BL1181 (SP_BL1181_) was PCR amplified from genomic DNA of *B. longum* strain (GenBank Accession Number: NC_004307.2) with the following primers: *Eco*RV-SP_BL1181_ (5′-G***GATATC***AAGGAGATATAAAAATGACGAACGTACGTGTGATCAAGCCCGC-3′) for the coding strand and *Nsi*I-SP_BL1181_ (5′-TG***ATGCAT***CTGCCTGGGCAGGCTGTGCCGAGCTGAACG-3′) for the complementary strand. The PCR product (132 pb) was subcloned into a pCR-TOPO kit (Invitrogen, Carlsbad, CA, United States) resulting in pCR-TOPO:*SP_BL1181_* (Table 1). Afterward, the DNA fragment containing SP_BL1181_ was obtained from this plasmid with *Eco*RV/*Nsi*I enzymes (Thermo Scientific) and cloned into purified backbone isolated from blunt-ended-*Afl*III/*Nsi*I-cut pBEST_Exp4_:IL-10 vector. The resulting plasmid pBEST_BL1181_:IL-10 (Figure 1) was established into *B. bifidum*.

For IL-10 detection, bacteria were grown until exponential phase (OD_600nm_ = 0.4–0.8) and the centrifuged culture resuspended (after one wash step with PBS) into the culture medium: MRS plus cysteine (500 mg/L) in order to apply different stresses: pH4, pH8, 42°C, NaCl (25 g/L) or bile salts (100 or 500 mg/L, Sigma-Aldrich, Saint-Quentin-Fallavier, France). Induction was performed in anaerobic conditions during 30 min. Culture extraction was performed as followings. Protein samples were prepared from 2 ml of induced cultures. After centrifugation (5 min, 5000 rpm), the cell pellet (C) and supernatant (S) were treated separately. The S samples were treated by filtration on 0.25 μm filter then frozen at -20°C. The C fraction was obtained after treatment in a PBS complemented with anti-protease 1× and lysozyme (100 mg/mL, Roche), at 37°C during 1 h and then sonicated (6 × 10 sec with a 30 s of timeout on ice). IL-10 concentration in S and C was assessed by ELISA (eBioscience, Paris, France) and normalized by the final OD_600nm_.

For western blot experiments, the C fraction was obtained as described for ELISA detection. The S samples were also treated by filtration on 0.25 μm filter plus a 50-fold concentration by the addition and precipitation of 10% trichloroacetic acid (TCA) during 15 min on ice and then centrifuged. Pellets were resuspended in NaOH. Protein concentration was determined by Bradford assay. Ten micrograms of protein were mixed with the blue gel-loading buffer and loaded in pre-casted gel (Bio-Rad). Proteins were then transferred on PVDF membrane and then blocked overnight in 5% milk in TBST. Membranes were stained with a primary rabbit polyclonal antibody against murine IL-10 (Abcam, ab9735) and a second goat HRP-conjugated against Rabbit IgG (BI2407, Abliance). Blots were reveled using Bio-Rad Kit.

### Detection of IL-10 *in vivo*

**FIGURE 2:**
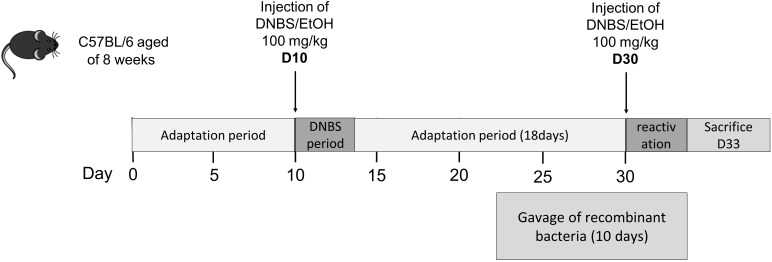
Experimental protocol for the mouse chronic low-grade inflammation model.

The two strains BS42:BEST_Exp4_:IL-10 and BS42:BEST_BL1181_:IL-10 were administered by oral gavage to 6 weeks old IL-10-KO mice during 11 days. To mimic the context of our standard experiment in WT mice (Figure 2), animals received one intra-rectal injection of a low dose of 2,4-DiNitroBenzene Sulfonic acid (DNBS, 2 mg/mouse), 7 days after the beginning of the gavage with bacteria. After sacrifice, colonic content and caecal content were collected. After being weighted, contents were diluted at 1/4 in PBS 150 mM pH7.2 with 1× anti-protease cocktail (Sigma-Aldrich, Saint-Quentin-Fallavier, France), and then placed 30 min at 4°C under mechanic agitation. Supernatants were collected after centrifugation and IL-10 was measured using ELISA (BioLegend, San Diego, CA, United States).

### DNBS-Induced Colitis Model

Male C57BL/6 mice (6 to 8-week-old) (Janvier, Le-Genest-Saint-Isle, France) were maintained at the animal care facilities of the National Institute of Agricultural Research (IERP, INRA, Jouy-en-Josas, France). Mice were housed under standard conditions for a minimum of 1 week before experimentation. All experiments were performed in accordance with European Community rules for animal care and were approved by COMETHEA C2EA-45 with authorization n°3445-2016010615159974.

For the induction of colitis, we used an adapted protocol from Martín (Martín et al., 2014; Figure 2) based on the intrarectal administration of DNBS. Briefly, mice (∼20 g) were anesthetized by intraperitoneal injection of 150 μL of 0.1% ketamine (Imalgene 1000, Merial) and 0.06% xylazine (Rompun). Then, a 4 cm-long catheter (Ecimed, Boissy-Saint-Léger, France) was attached to a tuberculin syringe, inserted into the colon and a dose of 100 mg/Kg of DNBS solution (Sigma-Aldrich, Saint-Quentin Fallavier, France) in 30% ethanol (EtOH) was injected intra-rectally to induce colitis in all group except control mice. Control mice (without colitis) received only 30% EtOH. Ten days after the so-called DNBS period, 1X10^9^ CFU of bacteria in PBS or PBS alone were intragastrically administered daily for 10 days (gavage period). The inflammation was reactivated 21 days after the first DNBS injection (recovery period) with a second injection of 100 mg/Kg of DNBS solution. Weight loss was monitored daily to assess the severity of colitis. Experiment was performed twice and mice were treated as follow: with ETOH-PBS (control mice without colitis, *n* = 16), with DNBS-PBS (colitis control group, *n* = 20), with *B. bifidum* BS42 wild-type strain (WT-BS42, *n* = 16), with *B. bifidum* BS42 harboring pBEST_Exp4_:IL-10 plasmid (BS42:BEST_Exp4_:IL-10, *n* = 16), and with *B. bifidum* BS42 harboring pBEST_BL1181_:IL-10 plasmid (BS42:BEST_BL1181_:IL-10, *n* = 16).

### Macroscopic Damage Scores

Macroscopic scores were recorded in the abdominal cavity using previously described system (Wallace and Keenan, 1990). Briefly, the macroscopic criteria (assessed on a scale from 0 to 9) include macroscopic mucosal damages such as ulcers, thickening of the colon wall, the presence of adhesions between the colon and other intra-abdominal organs, the consistency of fecal material (as an indicator of diarrhea), and the presence of hyperemia in colon (increase of blood flow).

### Histology on Colonic Samples

Fragment of colon were fixed in Formalin then dehydrated with ethanol 70%, and paraffin-embedded. Section of 3 μm-thick sections were stained with hematoxylin and eosin (H&E). As previously described (Cooper et al., 1993), colonic damage was scored according to architectural derangement, goblet cell depletion, edema/ulceration, and degree of inflammatory cell infiltration.

### Lipocalin-2 (LCN-2) Levels

Colonic contents were homogenized in FastPrep instrument with cold PBS. Supernatants were collected and stored at -80°C until analysis. LCN-2 was measured using R&D ELISA kit (R&D System, Minneapolis, MN, United States) and following manufacturer instruction.

### Cytokine Production by CD3/CD28-Stimulated Splenocytes

The day of sacrifice, spleens were removed and placed in RPMI medium for culture (Breyner et al., 2017). Briefly, spleens were crushed and filtered through a 70 μm nylon filter (BD, Le Pont-de-Claix, France) and were resuspended in RPMI (Lonza, Levallois-Perret, France) completed with 100 Unit of Streptomycin, Penicillin and 10% Fetal Calf Serum (FCS). Red blood cells were removed with buffered Blood Cell Lysis Buffer (Sigma-Aldrich, France). Plates were pre-coated with anti-CD3 and anti-CD28 antibodies, 4 μg/ml of each antibody (eBioscience) in PBS with 0.5% FCS. Splenocytes cells were adjusted to 2 × 10^6^ cells/ml per well and were cultured in 24-well plates at 37°C in a 5% CO2 and 95% air atmosphere. Supernatants were collected after 48 h of culture and were stored at -80°C until further analysis. Splenocytes culture supernatant levels of IL-4, IL-5 (Mabtech, Stockholm, Sweden), IL-6, IFN-γ (eBioscience), and IL-10 (BioLegend, San Diego, CA, United States) were quantified by ELISA, following manufacturer instructions.

### Statistical Analyses

Data were analyzed using GraphPad Prism (GraphPad Software Inc, San Diego, CA, United States) by non-parametric Kruskal–Wallis test followed by a Mann–Whitney test. A *P*-value of less than 0.05 was considered significant.

## Results

### Development of a New Expression System to Produce and Deliver IL-10 Cytokine in *Bifidobacterium bifidum*

To obtain a functional expression plasmid allowing *in vivo* stress-inducible heterologous protein expression in bifidobacteria, we first cloned the promoter from *B. longum dnaK* operon (Sanchez et al., 2005) into pLB270 plasmid (Table 1) resulting in pBEST_Exp4_:Nuc vector, replacing P*_nisA_* promoter by P*_dnaK_* promoter (Figure 1). We chose this promoter, because *dnaK* operon encodes heat-shock proteins partially involved in the adaptation and survival of some bacteria (such as Bifidobacteria) in the gut (Sanchez et al., 2005). In addition, in this genus, transcription of heat-shock proteins (and more particularly DnaK), are greatly enhanced by exposure to both bile salts and different pH.

**FIGURE 3:**
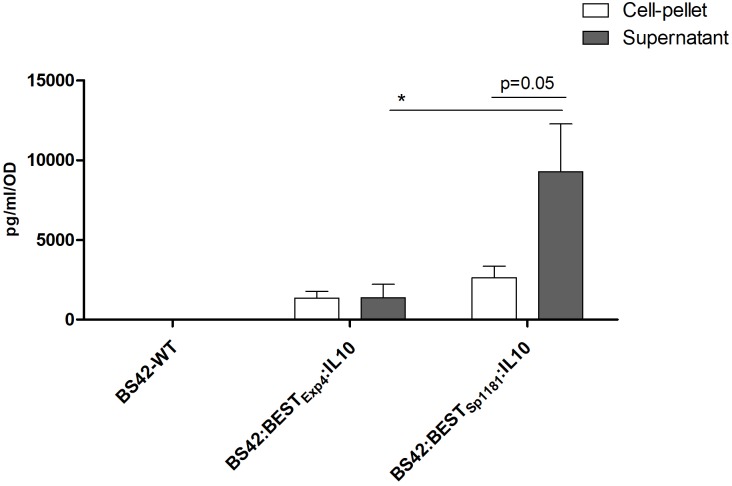
IL-10 production of recombinant Bifidobacteria BS42-WT, BS42:BEST_Exp4_:IL-10 and BS42:BEST_BL1181_:IL-10 grown to OD_600nm_ = 0.6–0.8 in MRS-cysteine at 37°C under anaerobic atmosphere. Cultures were washed once with PBS and placed in fresh medium, for 30 min at 37°C under anaerobic atmosphere. IL-10 was measured by ELISA in both cell-pellet (C) and supernatant filtered samples (S) (*n* = 3). Comparison between cell-pellet, supernatant and strains involved the non-parametric Mann–Whitney test. ^∗^*p* < 0.05.

**FIGURE 4:**
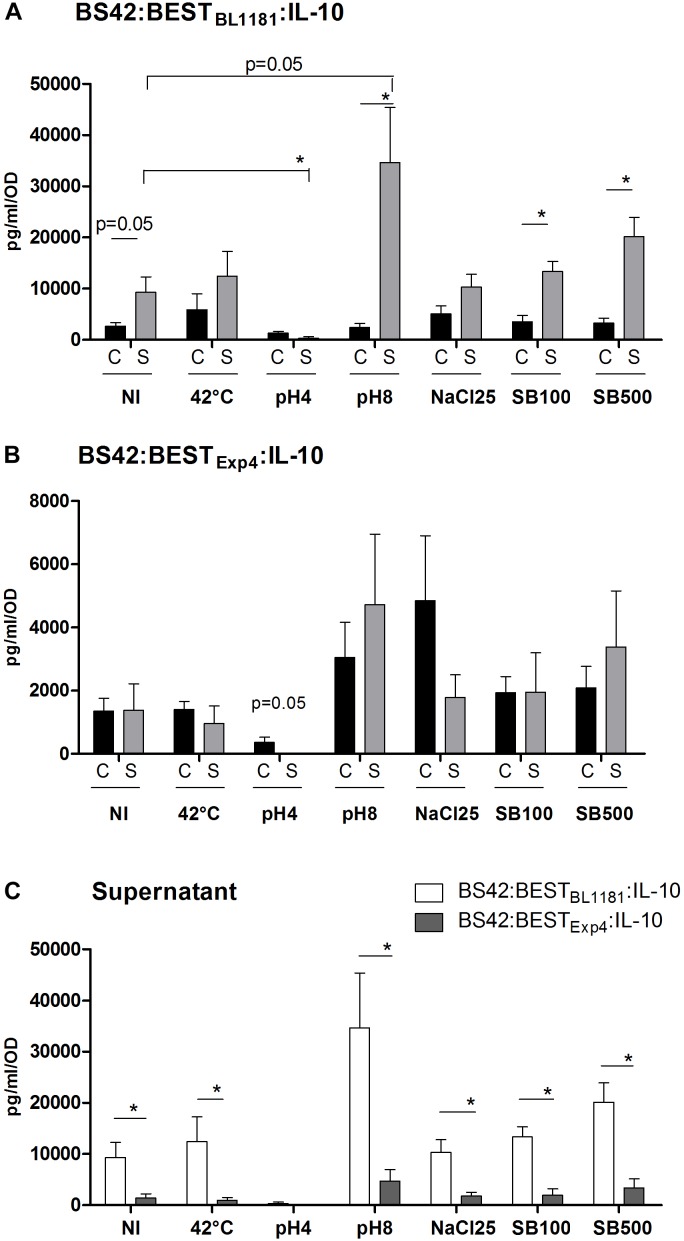
Effect of heat-shock, pH and bile salts on IL-10 production. BS42:BEST_BL1181_:IL-10 **(A)** and BS42:BEST_Exp4_:IL-10 **(B)** were cultured in MRS-cysteine broth under anaerobic condition until OD_600nm_ = 0.6–0.8. Cultures were washed once with PBS and re-cultured in fresh medium for 30 min at 37°C (except for heat-shock). **(C)** Comparison of IL-10 production in supernatant samples of the two recombinant strains. NI, non-induced (pH6); heat-shock: 42°C; acid shocks: pH4 and pH8; salt-shocks: NaCl25 = 25 g/L of NaCl; SB100 = 100 mg/L of bile salts; SB500 500 mg/L of bile salts. IL-10 was measured with ELISA in Cell-pellet and filtered Supernatant. Data (means plus SEMs) are the result of four separate experiments. Comparison involved the non-parametric Mann–Whitney test. ^∗^*p* < 0.05.

In order to validate the functionality of BEST system *in vivo, nuc* gene was replaced from this vector for murine IL-10 to obtain plasmid pBEST_Exp4_:IL-10. The resulting vector, pBEST_Exp4_:IL-10 (Figure 1), was established into *B. bifidum* BS42. As expected, no IL-10 was detected with the BS42 wild-type strain. As shown in Figure 3, *B. bifidum* BS42 harboring pBEST_Exp4_:IL-10 plasmid without any stress revealed IL-10 both in C and S samples reaching a maximal concentration of 2000 ng/L/OD. This phenomenon suggests a basal activity of the BEST system. To further explore if we can improve IL-10 secretion we replaced the SP_Exp4_ which is issued from *L. lactis* by a new one issued from a *B. longum* (SP_BL1181_). Our results show that there was no significant difference in C samples between the two recombinant strains; however, the constitutive production of IL-10 was sevenfold higher in S samples (9283 ± 2981 ng/L/OD) prepared from recombinant BS42:BEST_BL1181_:IL-10 compared to BS42:BEST_Exp4_:IL-10 strain: S (1377 ± 840 ng/L/OD) (*p* < 0.05) (Figure 3). Due to protein concentration and the sensitivity of western blot together with the fact that this cytokine is acid sensitive, only IL-10 produced by BS42:BEST_BL1181_:IL-10 in S samples was detected (Supplementary Figure S1). The *in vitro* production of IL-10 from recombinant *B. bifidum* BS42 harboring either pBEST_Exp4_:IL-10 or pBEST_BL1181_:IL-10 plasmid was studied under 6 different stress conditions during 30 min and compared to non-induced cultures in order to validate the inducibility of the BEST system (Figures 4A,B).

Regardless of the stress conditions, IL-10 concentration was higher in S fraction from BS42:BEST_BL1181_:IL-10 than BS42:BEST_Exp4_:IL-10 (*p* < 0.05, Figure 4C). Heat (42°C), NaCl, and bile salts stresses did not induce a significant difference in IL-10 concentrations neither in C nor S samples in BS42:BEST_BL1181_:IL-10 strain compared to non-induced condition (Figure 4A). In contrast, compared to non-induced conditions, IL-10 concentrations were significantly lower in S fraction at pH4 (*p* < 0.05) or higher at pH8 (*p* = 0.05). IL-10 amount was much higher in S than in C under pH8 and bile salt stress conditions (*p* < 0.05). Concerning the analysis in BS42:BEST_Exp4_:IL-10 strain (Figure 4B), nonetheless the stress condition, IL-10 levels were similar in both C and S samples even if IL-10 amount decreased in C at pH4 (*p* = 0.05). Finally, the two recombinant strains displayed a different IL-10 secretion profile.

### Detection of IL-10 Delivered *in vivo* in IL-10 KO Mice

Our IL-10 KO mice did not develop colitis: percentage of weight loss and macroscopic score were measured but no differences were observed (data not shown) between control and DNBS mice. Our results revealed that IL-10 could be detected in (i) ceacal samples of either 62.5% of mice (5/8) treated with BS42:BEST_BL1181_:IL-10 strain or 50% of mice (4/8) treated with BS42:BEST_Exp4_:IL-10, with a mean level of 0.759 and 0.554 pg/g, respectively (Supplementary Figure S2A); and (ii) colonic content samples of either 50% of mice (4/8) treated with BS42:BEST_BL1181_:IL-10 strain or 25% of mice (2/8) treated with BS42:BEST_Exp4_:IL-10, with a mean level of 0.280 and 0.097 pg/g, respectively (Supplementary Figure S2B). The presence of IL-10 in caecal and colonic content in IL-10 KO mice shows that the recombinant BS42:BEST_BL1181_:IL-10 and BS42:BEST_Exp4_:IL-10 are able to bring IL-10 *in vivo* within the digestive tract.

### Impact of IL-10 Delivery by Recombinant *B. bifidum* Using BEST System in a Low-Grade Inflammation Murine Model

**FIGURE 5:**
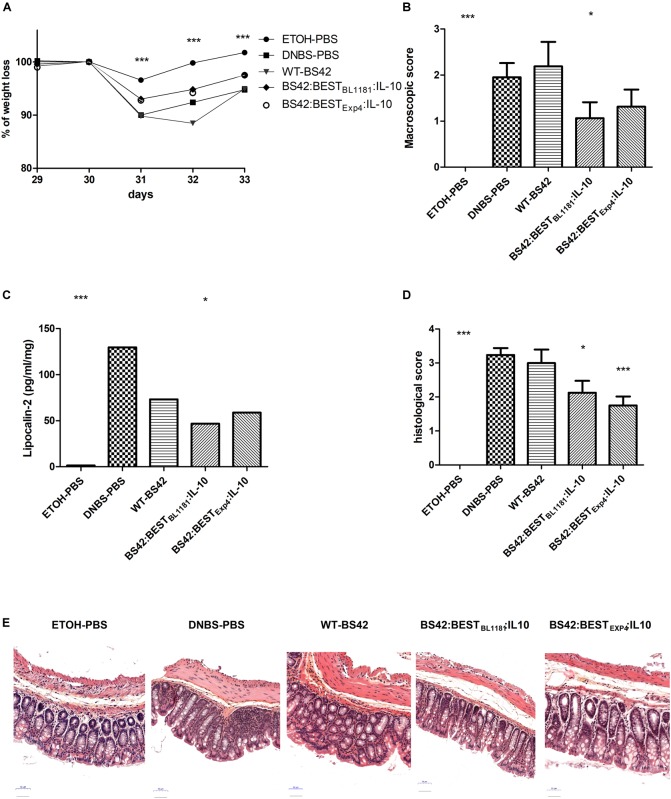
Impact of recombinant *B. bifidum* strains secreting murine IL-10 on a murine model of low grade inflammation induced by two rectal administrations of DNBS. Severity of the colitis reactivation was assessed by **(A)** change in body weight at day 33, **(B)** macroscopic scores and **(C)** fecal lipocalin-2 levels at day 34 and **(D)** histological score on colon and **(E)** Histological representation (H&E staining) (*n* = 2^∗^8 = 16 mice/group). Statistically significant differences are calculated relative to the DNBS group using non-parametric Mann–Whitney test. ^∗^*p* < 0.05, ^∗∗∗^*p* < 0.0001.

*In vivo* delivery of IL-10 through administration of BS42:BEST_BL1181_:IL-10 and BS42:BEST_Exp4_:IL-10 strains were then studied in a murine model of low-grade intestinal inflammation in wild type mice (Figure 2). As shown in Figure 5A, compared to ethanol, DNBS-treatment induced a weight loss (90.6 ± 0.94% at day 31, *p* < 0.001) after the second rectal injection and the next 3 days. Mice treated with IL-10-producing bifidobacterial strains tended to lose less weight, 93.06 ± 1.02% and 92.5 ± 1.18% for BS42:BEST_BL1181_:IL-10 and BS42:BEST_Exp4_:IL-10 groups, respectively, compared to the DNBS-PBS control group, (i.e., colitis control). Nevertheless, only the Ethanol-PBS group is significantly different compared to this colitis control group. The macroscopic score was significantly higher in the DNBS-PBS group compared to Ethanol-PBS group (*p* < 0.0001, Figure 5B). Mice treated with BS42 strain showed almost the same score as DNBS-PBS group. In contrast, BS42:BEST_BL1181_:IL-10 treatment significantly decreased this score (*p* < 0.05) and BS42:BEST_Exp4_:IL-10 induced a non-significant slight decrease in macroscopic score.

LCN-2, an early marker of inflammation, was measured in colonic content at day 33 (Figure 5C). The level of LCN-2 was significantly higher in the DNBS-PBS group compared to negative control (*p* < 0.0001). LCN-2 level was significantly lower with BS42:BEST_BL1181_:IL-10 group (*p* < 0.05), and, although not significant, tended to be decreased following treatment with BS42 and BS42:BEST_Exp4_:IL-10.

The histological score was significantly lower in BS42:BEST_BL1181_:IL-10 and BS42:BEST_Exp4_:IL-10 groups (respectively *p* < 0.05 and *p* < 0.0001) than in the DNBS-PBS group (Figures 5D,E).

**FIGURE 6:**
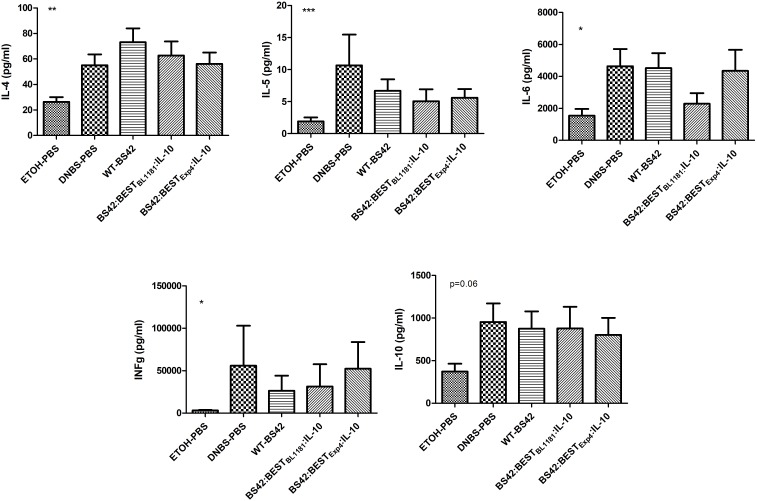
Impact of recombinant *B. bifidum* strains secreting murine IL-10 on the systemic T-helper balance. Cytokines secreted by splenocytes from each mice group, stimulated for 48h with CD3/CD28, were measured using ELISA (*n* = 2^∗^8 = 16 mice/group). Comparison was performed with the non-parametric Mann–Whitney test. Statistically significant differences were calculated relative to the DNBS group. ^∗^*p* < 0.05, ^∗∗^*p* < 0.001, ^∗∗∗^*p* < 0.0001.

The impact on systemic T-helper cells balance was determined by measuring IL-4, IL-5, IL-6, IFN-γ, and IL-10 cytokines in supernatant from mice splenocytes after 48H of CD3/CD28 re-stimulation (Figure 6). For all cytokines, there were significant differences between the colitis control group and the negative control group, confirming the inflammatory status of DNBS-PBS group. The intake of BS42:BEST_BL1181_:IL-10 slightly decreased IL-5, IL-6, and IFN-γ (not significant). However, there was no effect on IL-4 and IL-10. The intake of BS42:BEST_Exp4_:IL-10 as well as the BS42 tended to decrease the level of IL-5 and IFN- γ, with no effect on other cytokines.

## Discussion

In this work, we sought to construct an inducible expression system for bifidobacteria allowing *in vivo* delivery of proteins of health interest. For this, we developed a *Bifidobacterium* expression system (BEST) regulated by stress that we validated in *B. bifidum.* The functionality of BEST system was validated by cloning murine IL-10 cytokine and confirming both its correct expression and biological activity *in vivo* in a mouse model of low-grade intestinal inflammation. For the expression of IL-10 we confirm that the two recombinant *B. bifidum* strains were able to produce and secrete (even without any stress) the same IL-10 levels than those previously reported in recombinant bifidobacteria (Escogido et al., 2007; Khokhlova et al., 2010). For instance, a recombinant strain of *B. longum* was reported to produce 22 pg/ml of IL-10 (Escogido et al., 2007) whereas a recombinant strain of *B. breve* produced 1900 pg/ml of IL-10 (Khokhlova et al., 2010). In our study, we observed a constitutive production of IL-10 comparable or even greater (up to sevenfold) than those described with another plasmid, with an average yield of 1000 pg/ml with *B. bifidum* harboring pBEST_Exp4_:IL-10 plasmid and reaching 7000 pg/ml with *B. bifidum* harboring pBEST_SP1181_:IL-10. Moreover, at pH 8 (a stress condition under basic pH), we observed an IL-10 overproduction reaching 24000 pg/ml. This pH, which could reflect the pH of pancreatic juice when it is spilled into the duodenum, models a condition that *Bifidobacterium* can shortly encounter following oral administration. It shows the inductivity of our system as, in the large intestine where they are mainly located, *Bifidobacterium* will encounter a combination of factors and stresses which is difficult to reproduce *in vitro*. It is also interesting to note that pH8 can also stabilize IL-10. Indeed, it has been shown that at pH8.5, human IL-10 is under a highly stable dimer form (Syto et al., 1998). In the same study, the authors showed that IL-10 retained high activity between pH6 and 10. Below pH6.0, its activity decreased significantly and at pH2.5 only a small percent of IL-10 activity remained. A comparison analysis with commercial murine recombinant IL-10 allowed us to confirm that IL-10 produced by recombinant Bifidobacteria was stable in all the tested stresses conditions except at pH4 and lower (data not shown). In addition, pH4 can impact bifidobacteria growth. Except for some strains of *B. thermacidophilus*, bifidobacteria can survive (but not grow) at pH4.5–5 (Biavati and Mattarelli, 2015), this can explain that after 30 min at pH4, no IL-10 was detected in the culture medium.

To improve secretion, we included in the BEST system a new signal peptide issued from *B. longum*: SP_BL1181_. At pH8 (pancreatic juice), IL-10 secretion was 2.7-fold increased, confirming the interest of both SP_BL1181_ and P*_DnaK_* promoter. Concentrations of IL-10 in the C fraction were similar for our two recombinant BS42; however, a sevenfold higher concentration was observed in the S of BS42:BEST_BL1181_:IL-10 compared to that of BS42:BEST_Exp4_:IL-10. SP_Exp4_ allows only a small amount of IL-10 to be secreted compared to SP_BL1181._ This can be easily understandable by the bifidobacterial origin of this SP compared to SP_Exp4_ which is issued from *L. lactis*. Thus, BEST system using SP_BL1181_ seems to be an efficient system to secrete heterologous protein in *B. bifidum*. In addition, this SP (issued from *B. longum*) could also work in other *Bifidobacterium* strains, including *B. longum* itself; and although preliminary data suggest that SP_1181_ is functional in *B. longum* and *B. breve*, this remains to be confirmed.

To assess IL-10 *in vivo* delivery by recombinant bifidobacteria, we used IL-10 KO mice model. In these mice, following oral administration of the recombinant strains, we detected IL-10 in ceacal and colonic content confirming the production of IL-10 *in vivo*. Of note, these young IL-10 KO mice do not develop colitis despite the administration of DNBS, so we assess the biological efficacy of these recombinant bacteria on a well characterized low-grade intestinal inflammation model (Martín et al., 2014; Allain et al., 2016).

Several studies have reported beneficial effects of different strains of *B. bifidum* in a murine model of acute colitis (Preising et al., 2010; Alard et al., 2018). Our *B. bifidum* BS42 seems to have no beneficial impact as demonstrated on the murine model of low-grade intestinal inflammation. In our experiments, BS42:BEST_BL1181_:IL-10 had a greater beneficial effect on gut inflammation with a significant decrease in LCN-2 level than BS42. Despite not significant, BS42:BEST_BL1181_:IL-10 improved systemic inflammation as observed by the decrease in IL-6 and IFN-γ cytokines. Impact of BS42:BEST_Exp4_:IL-10 was less obvious confirming that IL-10 secretion is an advantage. In a previous work Martín et al. (2014) tested a recombinant strain of *L. lactis* producing murine IL-10 in the same model of low-grade intestinal inflammation and using the same dose of bacteria (*i.e.*, 1 × 10^9^ CFU/mouse) for the same administration period (11 days). If we compare the efficacy between the two recombinant strains, the effects of our BS42:BEST_BL1181_:IL-10 were more efficient regarding the weight evolution, the macroscopic and histologic scores and LCN-2 levels. In contrast, IL-10-producing *L. lactis* had a better effect on gut permeability and in cytokines levels. Despite each strain display its intrinsic properties, in the two cases, the presence of IL-10 in the intestine has beneficial effect on the low-grade intestinal inflammation model. It will be interesting to test in a near future a combination of these two recombinant strains producing IL-10 in different murine models of either IBS or IBD. Indeed, the ecological niche of *L. lactis* is the small intestine whereas for *Bifidobacterium* it is the colon and we can thus expect a synergic beneficial effect.

## Conclusion

In conclusion, we succeeded in the construction of a new Bifidobacteria Expression SysTem (BEST) that allowed *in vivo* IL-10 delivery by *B. bifidum.* Furthermore, the use of recombinant *B. bifidum* BS42 secreting IL-10 cytokine in a model of low-grade intestinal inflammation showed its ability to decrease local inflammation and confirmed therefore its potential for delivery of therapeutic molecules in the colon. Despite we consider our expression system is promising, more studies are needed, such as evaluate either daily or weekly bacteria administration which will allow us to determine the advantages of the higher persistency of this genus *versus* other recombinant bacteria (e.g., lactococci and lactobacilli).

## Author Contributions

LB-H and A-JW designed the study. AF performed the cloning. AM performed the *in vitro* characterization of IL-10 production. AM, FC, PR, and A-JW performed the animal experiments. AM performed all dosages. BR read the histological score. SG provided the analytical tools. AM, LB-H, and A-JW interpreted the data and wrote the manuscript. PL and M-JB provided the critical feedback, and helped to shape the research, analysis, and manuscript.

## Conflict of Interest Statement

The authors declare that the research was conducted in the absence of any commercial or financial relationships that could be construed as a potential conflict of interest.

## Footnotes

1 http://www.cbs.dtu.dk/services/SignalP/

## Supplementary Material

FIGURE S1 Western blot analyses of IL-10 in the C and S sample following or not 42°C induction.

FIGURE S2 *In vivo* production of IL-10. IL-10 KO-mice received daily BS42:BEST_Exp4_:IL-10 or BS42:BEST_BL1181_:IL-10, during 11 days by oral gavage. IL-10 were then measured in **(A)** caecal content and **(B)** colonic content by ELISA.
