# Supplementary figures and images for: A New Bifidobacteria Expression SysTem (BEST) to Produce and Deliver Interleukin-10 in Bifidobacterium bifidum
Source: Front Microbiol. 2018 Dec 21;9:3075. doi: 10.3389/fmicb.2018.03075 (PMC6308194; doi:10.3389/fmicb.2018.03075)

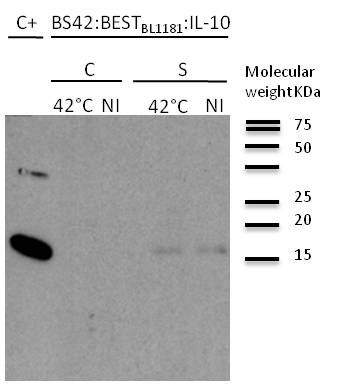

Supplement: FIGURE S1 — Western blot analyses of IL-10 in the C and S sample following or not 42°C induction. [file Image_1.JPEG]

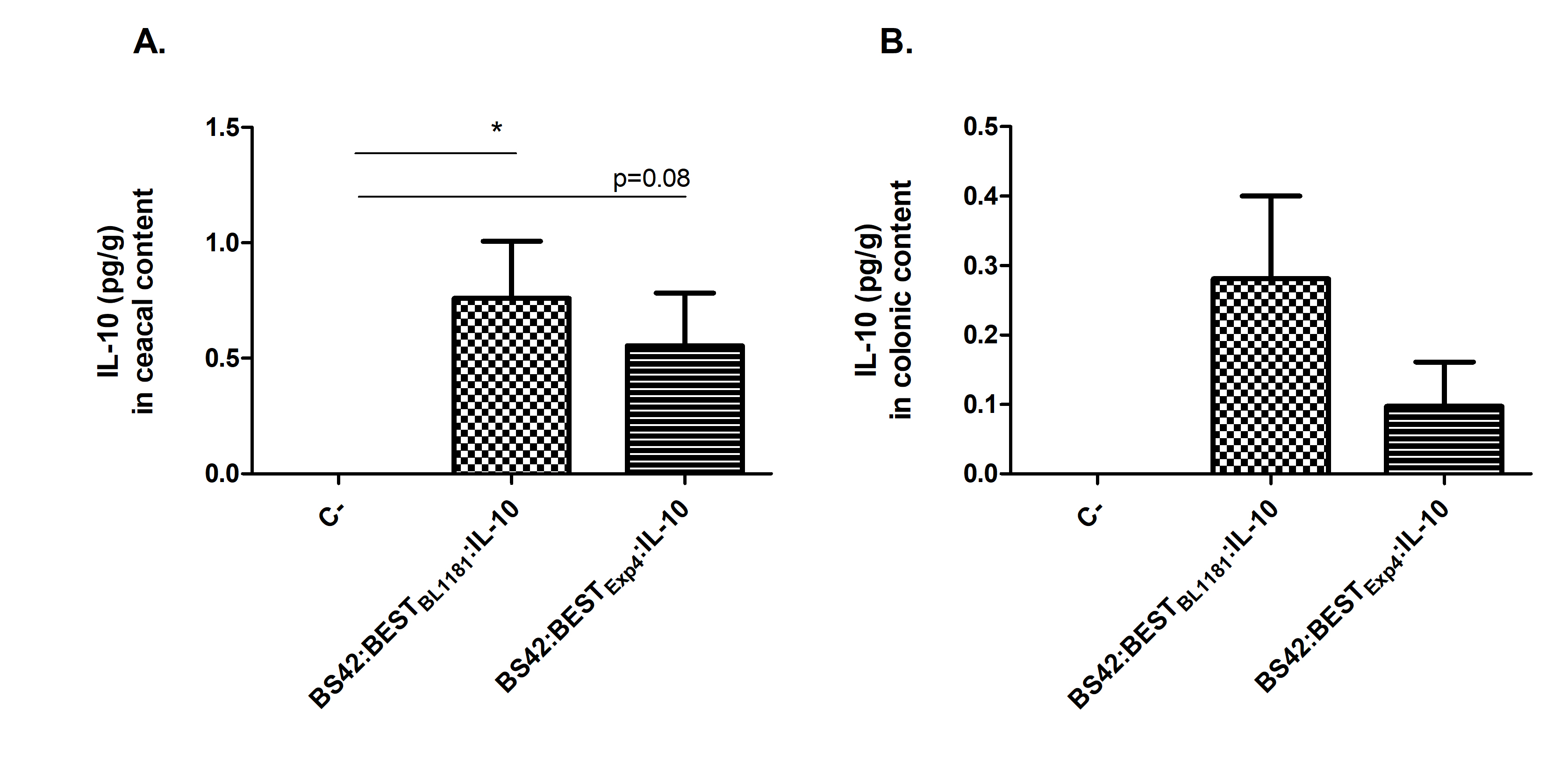

Supplement: FIGURE S2 — In vivo production of IL-10. IL-10 KO-mice received daily BS42:BESTExp4:IL-10 or BS42:BESTBL1181:IL-10, during 11 days by oral gavage. IL-10 were then measured in (A) caecal content and (B) colonic content by ELISA. [file Image_2.JPEG]
